# Poster Session I – A98 SOCIOECONOMIC FACTORS, ETHNICITY, AND EDUCATION IMPACT QUALITY OF LIFE AND MENTAL HEALTH IN PREGNANT IBD PATIENTS

**DOI:** 10.1093/jcag/gwaf042.098

**Published:** 2026-02-13

**Authors:** V Srikanth, V Premjeyanth, S Perera, K O’Connor, C Maxwell, T Zenlea, N Griller, S Vigod, V W Huang

**Affiliations:** Sinai Health, Toronto, ON, Canada; Sinai Health, Toronto, ON, Canada; Sinai Health, Toronto, ON, Canada; Sinai Health, Toronto, ON, Canada; Sinai Health, Toronto, ON, Canada; Women’s College Hospital, Toronto, ON, Canada; Sunnybrook Health Sciences Centre, Toronto, ON, Canada; Women’s College Hospital, Toronto, ON, Canada; University of Toronto, Toronto, ON, Canada

## Abstract

**Background:**

The global burden of inflammatory bowel disease (IBD) is rising, with many diagnoses occurring during reproductive years. IBD patients have a high prevalence of anxiety and depression, which negatively affects disease severity and quality of life (QoL). Socioeconomic (SE) factors may influence this relationship, as social barriers and vulnerabilities are associated with anxiety, depression, and poorer QoL. This study offers insights into SE factors in the context of IBD and pregnancy using patient-reported outcomes.

**Aims:**

To explore how SE factors affect self-reported anxiety, depression, and IBD-related QoL in pregnant IBD patients.

**Methods:**

These results are an extension of previously reported findings (PMC11807446). We conducted an anonymous, cross-sectional survey of pregnant IBD patients. Data collected included demographics, SE factors, IBD disease activity (modified Harvey Bradshaw Index (mHBI) for Crohn’s disease (CD) and Partial Mayo Score 6 (pMayo6) for ulcerative colitis (UC)), mental health scores for depression (PHQ-9) and anxiety (GAD-7), and IBD-related QoL (Short Inflammatory Bowel Disease Questionnaire (SIBDQ)). The Mann-Whitney U test was used to compare non-parametric, continuous variables between binary groups. A univariate logistic regression was conducted to compare income with comorbid anxiety and depression.

**Results:**

Sixty-nine surveys were analyzed (52.2% CD, 43.5% UC). The mean age was 33.87 ± 3.895 years. Of these, 60.9% had elevated mental health scores (PHQ-9 and/or GAD-7 ≥5): 5.8% had high PHQ-9 scores, 11.6% had high GAD-7 scores, and 43.5% had both. The median SIBDQ score was 57.0 (IQR 51.0–61.5), whereby 21.7% had impaired QoL (SIBDQ <50).

The ethnic distribution was primarily Caucasian (56.5%), followed by Asian (17.4%), and Other (26.1%). Asian participants had higher PHQ-9 (p = 0.008), lower QoL (p = 0.005), and a trend toward higher GAD-7 (p = 0.07). The majority held a university degree (53.6%); several had at least one advanced degree (31.9%), and a few reported having less than a university degree (14.5%). Those without a university degree had higher PHQ-9 scores compared to those with higher educational attainment (p = 0.002) and trended toward higher GAD-7 scores and lower QoL. Forty-nine participants had an annual household income over $100,000; fourteen had an income ranging between $50,000 and $100,000, and four below $50,000. The highest earners had lower GAD-7 (p = 0.014) and PHQ-9 (p = 0.003), and higher QoL (p = 0.027). They were also less likely to screen positive for comorbid anxiety and depression (OR = 0.126, 95% CI: 0.031–0.416, p = 0.001).

**Conclusions:**

In pregnant IBD patients, Asian ethnicity and lower educational attainment may be linked to poorer mental health and QoL, and higher income may be protective.

**Funding Agencies:**

University of Toronto: UT GE-HEP NEW INVESTIGATOR PILOT AWARD

